# Responding to safety concerns and chronic needs: trends over time

**DOI:** 10.1186/s13034-017-0200-5

**Published:** 2017-12-19

**Authors:** Barbara Fallon, Nico Trocmé, Joanne Filippelli, Tara Black, Nicolette Joh-Carnella

**Affiliations:** 1 Factor-Inwentash Faculty of Social Work, University of Toronto, 246 Bloor Street West, Toronto, ON M6S 3W6 Canada; 2 McGill University, 3506 University Street, Room#301, Montreal, QC H3A 2A7 Canada

## Abstract

**Background:**

For the past 20 years, the Ontario child welfare sector has made significant legislative and policy changes. Changes to legislation and policy can impact the public and sector’s response to child maltreatment and inform identified trends. Using an investigative taxonomy of urgent protection and chronic need this paper examines the shift in the nature of investigated maltreatment over time.

**Methods:**

Data from five cycles of the Ontario Incidence Studies of Reported Child Abuse and Neglect (1993, 1998, 2003, 2008 and 2013) were used. Provincial incidence rates were calculated by dividing the weighted estimates by the child population 15 years of age and under and then multiplying by 1000 in order to produce an annual incidence rate per 1000 children. Investigations were divided into urgent (severe physical harm, sexual abuse, neglect and physical abuse of children under 4) and chronic (risk only, exposure to intimate partner violence, emotional maltreatment, neglect and physical abuse of children four or over). Tests of statistical significance were calculated to assess changes in subtypes between cycles.

**Results:**

Between 1993 and 2013, the rate of child maltreatment related investigations completed in Ontario has increased from 20.48 per 1000 children to 53.27 per 1000 children. Overall there has been a decline in the incidence of urgent investigations from 9.31 per 1000 child maltreatment investigations in 1993 to 5.94 per 1000 maltreatment investigations in 2013. There has been a fourfold increase in the incidence of chronic investigations from 11.18 per 1000 child maltreatment investigations in 1993 to 47.33 per 1000 maltreatment investigations in 2013.

**Conclusion:**

The nature of child protection work using the urgent-chronic taxonomy shows a dramatic shift in the types of concerns identified without a corresponding shift in the way families are assessed for need. The provision of a forensic investigation to all families does not distinguish between urgent safety concerns and needs that may require prolonged engagement. Effective service provision requires more precision in our response to these diverse concerns.

## Background

Three children, ages 7, 9 and 11 are observed alone on public transit by a concerned citizen and a child welfare authority is contacted with an allegation that the children are not being provided with appropriate supervision. The next day, the same child welfare authority is contacted by a cousin of a woman who has been assaulted by her husband, worried that their teenager has witnessed the assault. The following day a teacher calls with a concern that a 2 year old sibling of one of her students is being left home alone when the mother walks her pupil to school in the morning. Each of these referrals to the child protection agency is judged to meet the threshold for investigation and while the number of “collaterals” interviewed may vary in each case, each family will receive a visit and an interview, the worker will complete a risk assessment and assess the child’s safety. There will be a determination about whether a child has been maltreated and whether the family needs ongoing child welfare services. The assessment of maltreatment varies across provinces and is determined by the clinical judgement of the investigating worker based on the balance of probabilities of whether the child has been maltreated.

Similar to other Canadian Provincial and Territorial statutes, safety and well-being are central and equal considerations within Ontario child welfare legislation [1]. Typically, a maltreatment-related concern for a child is reported to a child welfare authority. If the concern is deemed to be appropriate for an investigative response (i.e. screened-in), a series of decisions are made: whether or not to substantiate the concern, if ongoing child welfare services are required, and in rare cases, whether the child needs to be placed in out of home care. Investigative trends in Canada suggest a shift from urgent protection concerns to a greater focus on the consequences of family dysfunction on the development and well-being of children, or more chronic need [1]. For over 20 years, the Ontario child welfare sector has been undergoing significant legislative and policy changes. The approach to investigating a concern of abuse or neglect from the community about a child has largely remained unchanged. Using the investigative taxonomy of urgent protection and chronic need proposed by Trocmé and colleagues [1] and data from the five cycles of the Ontario Incidence Studies of Reported Child Abuse and Neglect [2–6], the purpose of this paper is to examine whether there has been a change in the type of maltreatment reported and investigated in Ontario from 1993 to 2013.

## The Ontario practice and policy context

The Ontario Incidence Study of Reported Child Abuse and Neglect (OIS) is the only source of aggregated provincial data on reported child maltreatment and provides an opportunity to explore possible changes in the incidence of reported child abuse and neglect over time. Reported child abuse and neglect in the province of Ontario doubled between 1998 and 2003; from a rate of an estimated 27.42 investigations per 1000 children to a rate of 53.56 per 1000 children in 2003. Since 2003, the rate of investigated maltreatment has remained consistent with the latest estimate in 2013 indicating that 53.27 investigations per 1000 children were conducted [5]. In 2008 a study examining the incidence of reported maltreatment in five provinces revealed that Ontario had the highest rate of maltreatment-related investigations (54.05 per 1000 children) and Quebec had the lowest rate of 13.19 per 1000 children [7].

Changes to legislation and policy can impact the public and sector’s response to alleged child maltreatment and inform identified trends. The increase in the incidence of investigations in Ontario is believed to be driven by the broadening of the child welfare mandate and the inclusion of children’s exposure to intimate partner violence, as well as those who were at future risk of maltreatment [8, 9]. Greater awareness of the adverse consequences of child maltreatment such as the increased likelihood of poor adult physical, mental health academic outcomes and economic hardship [10] may also be contributing to an increase in investigated reports [1].Child welfare practice and policy has been likened to a pendulum that swings between family-centred and more intrusive, child-centred service approaches [11]. Tragic events such as the death of a child can influence the orientation of the child welfare system. In Ontario, there were several high profile child deaths and coroner’s inquests in the 1990s that led to legislative changes and policy directives [8, 11] because there were concerns about the capacity of the child welfare sector to adequately protect children [8]. This pressure to improve the capacity of the child welfare system to respond led to a series of policy and legislative changes, which resulted in a shift to a more child-centred approach and a greater focus on immediate protection and safety of the child [9]. A new risk assessment model, comprised of three standardized decision-making tools (Ontario Eligibility Spectrum, Ontario Safety Assessment, and Ontario Risk Assessment) for all child welfare authorities was implemented in the province of Ontario in 1998 [12]. Risk assessment tools are designed to assist workers with the assessment of the future risk of maltreatment. The Ontario Child and Family Services Act (CFSA) was amended in March of 2000 and the definition of a child in need of protection was expanded [8]. Changes to the Act included clarifying its paramount purpose to promote the safety, well-being and best interests of children, lowering the thresholds for risk of harm and intervention, recognizing cases of neglect, clarifying the duty to report [8]. All of these factors are believed to have contributed to the increase in investigations in Ontario between 1998 and 2003 [1].

In 2006, further policy reform was initiated through the Ontario Child Welfare Transformation Agenda (Transformation Agenda), which included a more balanced approach to practice that protected children while also promoting their well-being and supporting families [13]. The Transformation Agenda promoted early intervention and permanency in child welfare [5, 13]. Several changes were made to Ontario child welfare as a result of the 2006 Transformation Agenda. In keeping with the increased focus on accountability, the Eligibility Spectrum was revised and new practice standards were introduced for child welfare across the service provision continuum, commencing from the receipt of the report and ending at the completion of the case. These standards further promoted customized responses and offering supports to families [5].

In 2009, the Commission to Promote Sustainable Child Welfare (the Commission) was established to better understand the impact of the Transformation Agenda, and to develop and implement further changes to improve the child welfare sector [14, 15]. A sustainable child welfare system was conceptualized as one that is adaptable to change, uses resources effectively, and has the ability to manage both short and long term demands [5, 14, 15]. Several changes emerged as a result of the commission, including the implementation of Child Protection Information Network (CPIN), a province-wide information system. CPIN has yet to be fully implemented across the province due to challenges associated with integrating different and independent information systems used by organizations to document case and financial management. In addition, there have been further revisions to Ontario’s Eligibility Spectrum and Standards in 2016 [16, 17]. Ontario will proclaim the Child, Youth and Family Services Act in 2018 in order to strengthen child welfare and improve outcomes for youth. It will raise the age of protection from 16 to 18 years, which is consistent with the United Nations Convention on the Rights of the Child [18]. The policy directions from the Transformation Agenda and the Commission have acted to highlight the importance of early intervention and support for at-risk children and families [9].

Despite significant policy and legislative changes that have occurred in Ontario over the last 20 years, there are concerns with traditional child welfare service models that emphasize child safety, and the ability of the sector to meet the complex, chronic needs of children and families served [1, 8, 11, 19]. Investigative trends in Canada have emphasized that differential or alternate response models are indicated [1, 11, 19]. There are a growing number of jurisdictions across North America that have implemented differential response models in child protection systems after several locations piloted this approach in the 1990s [20]. Differential response models typically assume a less adversarial approach with discrete pathways available to the family and the focus on the assessment of need traditional child welfare models emphasize more intrusive, forensic approaches [1]. Although several jurisdictions have implemented differential models to address the misalignment between client need and system response, there is no province-wide implementation of differential response models despite policy orientations that would support it. Child welfare systems need to refine their responses to meet the varied needs of children and families, and mobilize community resources [11].

The objective of this research is to determine rates at which child welfare authorities investigate maltreatment using the urgent-chronic taxonomy in Ontario to identify trends over time. A detailed analysis of investigative trends in Ontario by applying the urgent-chronic taxonomy can help to increase our understanding of how the child welfare system has been responding to population, policy and practice changes, and to its dual mandate of promoting safety and well-being.

## Methods

Data from the five OIS cycles were analyzed to explore trends in the investigative taxonomy of urgent-chronic need. Each of the five cycles of the Ontario Incidence Study of Reported Child Abuse and Neglect (OIS) utilized a multi-stage sampling design [9]. The first stage of sampling included the selection of a representative sample of child welfare sites from a sampling frame that includes all mandated child welfare organizations in Ontario. Secondly cases that were opened in the study sites during the 3-month period from October 1 to December 31 in the year the study took place were selected. A 3-month sampling period is considered optimal for high participation rates and good compliance with study procedures [5]. Investigations were evaluated by study staff to ensure that they met the OIS definitions of maltreatment. In 2008 and 2013 the definition of maltreatment was expanded to include risk of future maltreatment. The 1993, 1998 and 2003 OIS cycles did not track cases where there was no specific maltreatment concern alleged or suspected during the course of the investigation or “risk only cases”. Risk only cases where included beginning in 2008 and collected information about investigations in which there was no specific allegation of an incident of maltreatment, but rather the future risk of maltreatment was assessed.

The final stage of sampling includes identifying children investigated as a result of maltreatment concerns. In each of the five OIS cycles, estimates of the provincial annual rates of maltreatment investigations in Ontario were derived by applying a regionalization and annualization weights. Estimates for each of the cycles do not include incidents not reported to Ontario child welfare authorities, cases that were screened out and were not fully investigated, new reports on cases that were opened, and cases that were only investigated by police [5]. For greater detail on the design and weighting procedures, see the methods chapters specific to each of the five study cycles [2–6]. Please see Table 1 for the number of agencies, investigations and estimates of child maltreatment investigations completed across the OIS cycles.

**Table 1:** Sites and sample sizes for the Ontario Incidence Study of Reported Child Abuse (OIS) Cycles

	OIS-1993	OIS-1998	OIS-2003	OIS-2008	OIS-2013
Site selection (sample/total)	15/51	13/53	16/53	23/53	17/46
Case selection	1898	2193	4175	4415	3118
Investigated children	2447	3053	7172	7471	5265
Estimate of child maltreatment investigations	46,683	64,658	128,108	128,748	125,281

Data for each of the OIS cycles is collected directly from investigating child welfare workers in each of the sampled organizations using a three-page standardized data collection instrument, the maltreatment assessment form. This instrument is completed at the conclusion of the investigation and collects clinical information that is routinely gathered by child welfare workers during the course conducting the investigation, including caregiver, child, case, and short-term service dispositions. In 2008, the maltreatment assessment form was amended to include investigations that were conducted which focused not on an event of maltreatment that alleged or suspected but rather assessed the risk to the investigated child of future maltreatment or *risk only* investigations.

## Analysis plan

SPSS Statistic version 23 was used to conduct the analyses. Provincial incidence rates were calculated by dividing the weighted estimates by the child population 15 years of age and under and then multiplying by 1000 in order to produce an annual incidence rate per 1000 children.

The population for Ontario children is based upon the appropriate Census data for the study cycle. The Census is conducted by Statistics Canada every 5 years.

First, the overall investigation rate for investigations in Ontario in each of the five OIS cycles (1993, 1998, 2003, 2008, and 2013) was compared over time. Next, we examined the change in the rates of investigations using the urgent-chronic taxonomy across cycles of the OIS. Investigations were classified as urgent or other maltreatment-related investigations or assessments (i.e. chronic need) by using the taxonomy developed by Trocmé and colleagues [1]. Classifications were by the primary form of maltreatment, child age and the presence of severe harm requiring medical treatment. Investigations were assessed as urgent protection if the child was younger than 4 years of age and was investigated for neglect of physical abuse, if the primary concern was sexual abuse, or if the child had sustained physical harm and required subsequent medical treatment. Urgent protection investigations were compared to other investigations or assessments.

Statistical significance was calculated to examine whether there had been a change in the incidence from the previous OIS cycle. Tests of significance were produced using WesVar 5.1 software.

## Results

**Fig. 1:**
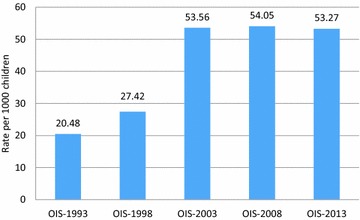
Rate of child maltreatment investigations in Ontario in 1993, 1998 and 2003 and child maltreatment investigations and risk of future maltreatment investigations in Ontario in 2008 and 2013

Figure 1 presents the incidence of reported maltreatment investigations in Ontario for each of the five cycles, between 1993 and 2013 in Ontario. Between 1993 and 2013, the rate of child maltreatment related investigations completed in Ontario since 1993 has gone from 20.48 per 1000 children to 53.27 per 1000 children. The incidence of investigations did not significantly change between 2003, 2008 and 2013.

Table 2 presents a classification of investigations by the urgent and chronic taxonomy from 1993, 1998, 2003, 2008 and 2013.

**Table 2:** Incidence of urgent protection and chronic investigations and assessments in Ontario in 1993, 1998, 2003, 2008 and 2013

Type of investigation	OIS-1993			OIS-1998			OIS-2003			OIS-2008			OIS-2013		
	#	Rate per 1000	%	#	Rate per 1000	%	#	Rate per 1000	%	#	Rate per 1000	%	#	Rate per 1000	%
Urgent protection investigation															
Severe physical harm	890	0.41	2	2400	1.02*	4	1908	0.80	1	1729	0.73	1	1445	0.61	1
Physical abuse < 4	3472	1.59	8	3399	1.44	5	4283	1.79	4	2294	0.96	2	2704	1.15	2
Sexual abuse	11,315	5.17	25	6079	2.58*	9	6661	2.79	5	4557	1.91*	4	4212	1.79	3
Neglect < 4	4702	2.15	10	6059	2.57	9	10,197	4.27	8	7552	3.17	6	5603	2.38	4
Total urgent protection	20,379	9.31	45	17,937	7.61	28	23,049	9.64	18	16,132	6.77	13	13,964	5.94	11
Chronic investigations and assessments															
Physical abuse ≥4	14,930	6.82	33	19,207	8.15	30	31,777	13.29	25	20,142	8.46	16	21,804	9.28	17
Neglect ≥4	7546	3.45	17	15,795	6.70*	24	30,252	12.65*	24	20,493	8.60	16	20,291	8.64	16
Emotional maltreatment	1995	0.91	4	5076	2.15***	8	18,409	7.70***	14	8039	3.37*	6	10,566	4.50	8
Exposure to IPV	–	–	0	6607	2.80	10	24,564	10.27***	19	22,219	9.33	17	31,210	13.28	25
Overall risk	–	–	0	–	–	0	–	0	0	41,723	17.52	32	27,330	11.63	22
Total other investigations	24,471	11.18	55	46,685	19.81	72	105,002	43.94**	82	112,616	47.28	87	111,201	47.33	89
Total	44,850	20.48	100	64,622	27.42	100	128,051	53.56*	100	128,748	54.05	100	125,165	53.27	100

* p < 0.05, ** p < 0.01, *** p < 0.001

### Urgent investigations

As shown in Table 2, the rate of severe physical harm is similar from 1993 to 2013. In 1998 the rate of severe physical harm rose to 1.02 per 1000 investigations from .41 per 1000 investigations in 1993; however, the proportion of cases with documented physical harm is consistently low, ranging from 1 to 4% of investigations. Similarly, the rate of investigated physical abuse for children under the age of 4 has remained consistent over time from a low of 0.96 per 1000 child maltreatment investigations in 2008 to a high of 1.79 in 2003. Reported sexual abuse investigations have declined with the largest reduction in investigations between 1993 and 1998: in 1993 the rate of investigated sexual abuse was 5.17 per 1000 child maltreatment investigations; in 1998 the incidence of reported sexual abuse was 2.58 per 1000 investigations (p < 0.01). For neglect investigations of children under the age of four, the rate of investigations nearly doubled between 1998 and 2003 from 2.57 per 1000 child maltreatment investigations in 1998 to 4.27 per 1000 child maltreatment investigations in 2003, although this increase was not statistically significant.

Overall there has been a decline in the incidence of urgent investigations from 9.31 per 1000 child maltreatment investigations in 1993 to 5.94 per 1000 maltreatment investigations in 2013. As a proportion of all investigations, those of an urgent nature have declined from 45% of all investigations in the OIS-1993 to 11% of all investigations in the OIS-2013.

### Chronic investigations

The nearly doubling of the rate of child maltreatment investigations in Ontario between 1998 and 2003 is reflected in some of the trends in the subtypes of chronic investigations. Physical abuse investigations for children 4 years of age and older went from a rate of 8.15 per 1000 investigations in 1998 to 13.29 per 1000 investigations in 2003, returning to 8.46 per 1000 investigations in 2008 and 9.28 per 1000 investigations in 2013. A very similar pattern is seen in the rate of neglect investigations for children 4 years of age and older.

In 1993, emotional maltreatment was investigated in only .91 per 1000 investigations. In 1998 there was a twofold increase to 2.15 per 1000 investigations and the rate of investigated emotional maltreatment continued to increase in 2003 to 7.70 per 1000 investigations. In 2008, there was a statistically significant decline in investigated emotional maltreatment to 3.37 per 1000 investigations. In 2008 one in three investigations focused on risk of future maltreatment (17.52 per 1000 investigations). In 2013 this proportion declined to one in five investigations (11.63 per 1000 investigations), although this decline was not statistically significant. Children exposed to intimate partner violence were not identified in 1993; in 2013 it had the highest incidence of investigations, 13.28 per 1000 investigations.

Overall there has been a fourfold increase in the incidence of chronic investigations from 11.18 per 1000 child maltreatment investigations in 1993 to 47.33 per 1000 maltreatment investigations in 2013. As a proportion of all investigations, those of a chronic nature have increased from 55% of all investigations to 89% of all investigations.

## Discussion

The rate of child maltreatment related investigations completed in Ontario since 1993 has gone from 20.48 per 1000 children to 53.27 per 1000 children. Two decades of policy and legislative changes have resulted in a dramatic change in the type of situation that child protection workers are routinely faced with. The overall increase in child maltreatment investigations in Ontario is difficult to interpret; for instance, the rate of child homicides has remained fairly consistent in Ontario for several decades [21]. Ontario’s child population has varied little from 1993 to 2013. The child population increased by 8% between 1991 and 1996 but has remained stable from 1996 to present day [22–26]. The percentage of children in poverty has also remained consistent since the 1990s —approximately 15% of children in Ontario live below the poverty line [27], while unemployment rates have decreased from just over 8% in 2012 to 5.8% in January 2017 [28]. The incidence of reports to child welfare organizations for a child maltreatment related concern did not change after the 2008 Great Recession.

Urgent cases are investigations where the child has sustained harm serious enough to require medical treatment; there is an allegation of sexual abuse or there is a concern of physical abuse or neglect for a child under the age of four. The rate of urgent cases has both declined by nearly half (from 9.31 per 1000 investigations in 1993 to 5.94 investigations in 2013) and in proportion to the overall composition of investigations. The findings in this paper of a decline in reported sexual abuse investigations is consistent with a steady decline in sexual abuse since the 1990s in the United States [29, 30] and the Canadian incidence studies, although statistics from victimization surveys and police databases do not support the finding that child sexual abuse is in decline [31]. Severe physical harm is consistently noted in only a small proportion of investigations. In 1993 urgent investigations were almost half the work of an Ontario child protection worker, in 2013 a worker assessed an urgent investigation in one of every ten cases.

The Ontario child welfare legislation specifically includes situations where a child has been harmed or is at risk of being harmed, consistent with an emerging body of research that show chronic, unaddressed maltreatment results in behavioural, emotional, cognitive and health issues [1, 19, 32, 33]. This inclusion is reflected in some of the dramatic increases in the subtypes of chronic investigations. Strikingly, the assessment of future risk of maltreatment and investigations focusing on exposure to intimate partner violence was nearly half the investigative work of the child welfare system in Ontario in 2013. It is difficult to disentangle the complexity of issues by categorizing investigations through one subtype. In many investigations, intimate partner violence, mental health, substance use, poverty and few social supports co-occur but may not be the primary focus of the investigation [32, 34]. These families and children are at no less risk for dramatically poor outcomes than families and children involved in urgent investigations, but the nature of the concern requires less focus on the immediate physical safety of the child and more focus on the long term effects of family related problems. Standardized decision making tools have been helpful in assisting investigating workers in determining whether a child is at future risk of maltreatment. The results of this study demonstrate that one of the most important functions a child welfare worker can perform is the assessment of family functioning and need not only to mitigate the future risk of maltreatment but to align clinical and developmental concerns with the appropriate available services both within a child welfare agency but also in the broader community. The provision of effective child welfare services not only require the identification of the complex clinical needs of the children and families identified to the child welfare system but the ability to provide assistance and programs that are evidenced informed. Fundamentally, at present, there is a disconnect between the identified need and response.

Despite the changing nature of the type of maltreatment reported and investigated in Ontario, the response by the system is nearly identical to the investigation procedures. The concerns with a traditional or child protection response to maltreatment often considered adversarial and intrusive has led some jurisdictions in the United States and Canada to develop and implement a formal differential or alternative response to children and their families [20]. This response was aimed at aligning family needs to service through a detailed assessment. In Ontario, differential response has been implemented as the possibility that a worker can investigate the allegation in a more inclusive or customized manner. Nonetheless, the system still requires the use of the same tools and a determination about whether there is a protection concern. There is no discrete pathway in legislation, policy or practice to the provision of services without an investigation. If a child is determined to be in need of child welfare services, the case must be opened for protection services.

### Limitations

The OIS collects information directly from the investigating worker and the data collected are not independently verified. The study only examines the case until the point of initial assessment—the data are not able to describe the longer term impact of the events described. The data do not include children who are only reported to the police, known to a community member or never disclose their abuse or neglect. There have been procedural and study definitional changes over time that reflect changes in legislation and procedures, in particular allowing workers to describe investigations as *risk only* in 2008 makes comparisons across cycles challenging. For example, the variation in reported emotional maltreatment investigations is likely to have been impacted by the inclusion of risk only investigations in 2008 as the chronic nature of these situations may be similar.

## Conclusion

Data from the Ontario Incidence Studies of Reported Child Abuse and Neglect describe a child protection system that expanded rapidly between 1998 and 2003 and since then has consistently investigated five and a half percent of children 15 years of age and under for a child maltreatment-related concern. The nature of child protection work using the taxonomy developed by Trocmé and colleagues [1] shows a dramatic shift in the types of concerns identified without a corresponding shift in the way families are assessed for need. The provision of a forensic investigation to all families does not distinguish between urgent safety concerns and needs that may require prolonged engagement. Responding to the safety and longer term issues for the latency age children who are unsupervised on a bus, the toddler left alone in an apartment and the youth who witnesses his father assault his mother requires a system that can be attenuated to their distinct needs that aligns and advocates for services and supports within the child welfare system. Indeed, chronic conditions are ones that over-time will require the most extensive response not only from child welfare but from other related sectors. Effective service provision requires more precision in our response to these diverse concerns.

## Abbreviation

OIS: Ontario Incidence Study of Reported Child Abuse and Neglect
